# Endothelial Barrier Integrity Is Disrupted *In Vitro* by Heme and by Serum From Sickle Cell Disease Patients

**DOI:** 10.3389/fimmu.2020.535147

**Published:** 2020-12-14

**Authors:** Vanessa Araujo Gomes Santaterra, Maiara Marx Luz Fiusa, Bidossessi Wilfried Hounkpe, Francine Chenou, Wouitchekpo Vincent Tonasse, Loredana Nilkenes Gomes da Costa, Diego Garcia-Weber, Igor de Farias Domingos, Franciele de Lima, Ivanio Teixeira Borba-Junior, Aderson da Silva Araújo, Antonio Roberto Lucena-Araújo, Marcos André Cavalcante Bezerra, Magnun Nueldo Nunes dos Santos, Fernando Ferreira Costa, Jaime Millán, Erich Vinicius De Paula

**Affiliations:** ^1^ School of Medical Sciences, University of Campinas, Campinas, Brazil; ^2^ Department of Biomedicine, Federal University of Piaui, Parnaiba, Brazil; ^3^ Centro de Biologia Molecular Severo Ochoa, Consejo Superior de Investigaciones Cientificas, Universidad Autonoma de Madrid, Madrid, Spain; ^4^ Genetics Postgraduate Program, Federal University of Pernambuco, Recife, Brazil; ^5^ Department of Clinical and Toxicological Analysis, Federal University of Rio Grande do Norte, Natal, Brazil; ^6^ Department of Internal Medicine, Hematology and Hemotherapy Foundation of Pernambuco (HEMOPE), Recife, Brazil; ^7^ Hematology and Hemotherapy Center, University of Campinas, Campinas, Brazil

**Keywords:** endothelial barrier, heme, sickle cell disease, electric cell-substrate impedance sensing, danger-associated molecular pattern, hemopexin

## Abstract

Free extracellular heme has been shown to activate several compartments of innate immunity, acting as a danger-associated molecular pattern (DAMP) in hemolytic diseases. Although localized endothelial barrier (EB) disruption is an important part of inflammation that allows circulating leukocytes to reach inflamed tissues, non-localized/deregulated disruption of the EB can lead to widespread microvascular hyperpermeability and secondary tissue damage. In mouse models of sickle cell disease (SCD), EB disruption has been associated with the development of a form of acute lung injury that closely resembles acute chest syndrome (ACS), and that can be elicited by acute heme infusion. Here we explored the effect of heme on EB integrity using human endothelial cell monolayers, in experimental conditions that include elements that more closely resemble *in vivo* conditions. EB integrity was assessed by electric cell-substrate impedance sensing in the presence of varying concentrations of heme and sera from SCD patients or healthy volunteers. Heme caused a dose-dependent decrease of the electrical resistance of cell monolayers, consistent with EB disruption, which was confirmed by staining of junction protein VE-cadherin. In addition, sera from SCD patients, but not from healthy volunteers, were also capable to induce EB disruption. Interestingly, these effects were not associated with total heme levels in serum. However, when heme was added to sera from SCD patients, but not from healthy volunteers, EB disruption could be elicited, and this effect was associated with hemopexin serum levels. Together our *in vitro* studies provide additional support to the concept of heme as a DAMP in hemolytic conditions.

## Introduction

Heme is a ubiquitous molecule present in almost all forms of life, that is normally found conjugated to hemoproteins such as hemoglobin (Hb), the most abundant pool of heme in mammals. However, despite its importance in biological pathways such as oxygen transportation, several lines of evidence demonstrate that free extracellular heme (FEH) can also be toxic to cells, a concept supported by the selection of extremely effective extracellular scavenging mechanisms (e.g., hemopexin) that preclude the circulation of FEH (1–3).

The toxicity of FEH is particularly important for the pathogenesis of conditions associated with increased intravascular hemolysis (hence, high free Hb levels), since oxidation of free Hb has been shown to increase the rate of heme release to the extracellular space (4). FEH toxicity can be caused by direct (i.e., intercalation of heme in cellular membranes) or indirect (i.e., immune-mediated) mechanisms, and in regard to the latter, several studies demonstrated that heme can activate a myriad of innate immunity compartments such as TLR4-dependent pathways (5), neutrophil/neutrophil extracellular trap release (6, 7), complement (8, 9), inflammasomes (10), and hemostasis (11–13). Together, these studies support the notion that heme can act as a danger-associated molecular pattern (DAMP) in diseases characterized by high hemolytic rates such as malaria, sepsis, hemolytic uremic syndrome and sickle cell disease (SCD), where FEH could trigger and/or contribute to the underlying inflammatory response (14–17).

Localized endothelial barrier (EB) disruption is an important and finely regulated part of innate immune response that allows circulating leukocytes to reach inflamed tissues (18). However, deregulated EB disruption can lead to widespread microvascular hyperpermeability and secondary tissue damage (19, 20). While this possibility is more evident in conditions such as sepsis-associated acute lung injury (ALI) and cerebral malaria, studies in mice models of SCD recently demonstrated that EB disruption could contribute to the pathogenesis of acute chest syndrome (ACS), a form of ALI that figures among the main causes of death in SCD (21). Moreover, these studies demonstrated that FEH can cause a severe and fatal form of ALI in SCD mice, preceded by congestion and edema of alveolar spaces (22). In fact, the barrier-disrupting effects of heme have been demonstrated more than 15 years ago (23), and were recently confirmed in studies using endothelial cell cultures stimulated by FEH by independent groups, using different experimental designs (24–26). However, to the best of our knowledge no study evaluated the effect of sera from SCD patients with varying levels of heme on EB integrity. Moreover, one of the caveats of studying the pathological relevance of heme refers to the unstable nature of FEH in biological systems due to its fast binding kinetics to circulating proteins (such as hemopexin and albumin), allowing some authors to question the concept that heme can act as a DAMP in living organisms (27). Here we explored the effect of heme on human endothelial cell monolayers in experimental conditions designed to address some of these caveats, using a robust functional assay (28, 29) that has been previously used in studies of EB function in other inflammatory conditions (30, 31).

## Materials and Methods

### Reagents, Antibodies, and Cells

Heme was obtained from Frontier Scientific (Frontier Scientific, USA) and TNFα from Biolegend (Biolegend, USA). Endothelial basal medium (EBM-2), endothelial cell growth medium supplement (EGM-2), and primary human umbilical vein endothelial cells (HUVEC) were obtained from Lonza (Walkersville, MD, USA). Rabbit monoclonal anti–VE-cadherin antibody was from Cell Signaling Technology (Boston, MA, USA), and Alexa fluor 555-phalloidin was obtained from Life Technologies (Gaithersburg, MD, USA).

### Cell Culture

HUVECs were grown in fibronectin (10 µg/ml) pre-coated 75 cm² flasks in EBM-2 medium supplemented with 2% fetal bovine serum and with EGM-2, at 37°C in an atmosphere of 5% CO_2_/95% air, as previously described (32). Medium was replaced every 48 h until confluence (approximately 80%) was reached. All experiments were performed in HUVECs between passages 3 to 5.

### *In Vitro* Evaluation of Endothelial Barrier Function

EB integrity was measured using ECIS, an electric cell-substrate impedance sensing system (ECIS Zθ, Applied BioPhysics, Troy, NY) (28, 29, 33). Cells were seeded (2.5 × 10^5^ cells/well) and grown to confluency on fibronectin-coated (10 µg/ml) eight-well arrays (8WE10, Applied BioPhysics, Troy, NY) containing interdigitated gold electrodes, specific for this system. The system is based on the application of a weak alternating current through the electrode array, and on the continuous measurement of the ability of the cell monolayer to impede the movement of electrons between adjacent endothelial cells. This resistance is expressed by the parameter R (resistance), a component of the impedance measured by ECIS (34). As previously shown, at low frequencies the movement of current between cells is mostly restricted by the presence of intercellular junctions (35, 36). Endothelial cells were normally seeded 48 h before experiments and R was recorded after 48 h. Only wells with R > 1,500 ohms and stable impedance/resistance readings were used. Before stimulation, resistance was continuously monitored for 2 h, to confirm EB stability represented by a plateau in the R curve. Stimuli were then added to wells under continuous impedance/resistance monitoring, for the time indicated in each experiment. A baseline R value was recorded immediately prior to the addition of each stimuli, and results were then expressed as a ratio from baseline resistance (normalized R). The lower the normalized R value, the higher the magnitude of EB disruption of cell monolayers.

### Stimulation of Endothelial Cells

Heme was diluted to an initial working concentration of 5 mM in NaOH 0.1 M. This solution was filtrated through a 0.22-µm filter and immediately used in experiments, at concentrations from 5 to 100 µM (diluted in serum free EBM-2 medium). Of note, the final concentration of NaOH in these solutions varied from 0.01 to 0.2 µM respectively. NaOH solutions with concentrations equivalent to those used to in heme dilutions of 30, 50, and 100 µM were used as negative controls (vehicle), as detailed in figure legends. TNFα was diluted in serum free EBM-2 medium and immediately added to cell monolayers. Sera from patients or healthy volunteers were diluted (20% v/v) in EBM-2, as previously described (37). The protein concentration of final sera preparations corresponded to 20% of serum total protein concentration from each subject (shown in **Table S1**) and varied from varied from 1.24 to 1.98 g/dl in patients, and 1.22 to 1.54 g/dl in healthy volunteers.

### Patients and Healthy Volunteers

The study was performed in accordance with the Declaration of Helsinki and approved by the local Institutional Review Boards of both HEMOPE and University of Campinas (protocols 510.517 and 3.291.418 respectively). Written informed consent was obtained from all subjects or their legal representatives prior to enrollment. The study population consisted of 20 patients with SCD (all with sickle cell anemia—HbSS) followed at HEMOPE Foundation (Recife, PE, Brazil) and 10 healthy volunteers from the same geographic region and ethnic background. These individuals were part of a cohort from an ongoing collaborative study aimed to investigate the association of haptoglobin polymorphisms with markers of endothelial activation in SCD. Patients were selected from this cohort based on pre-determined serum heme levels measured by a colorimetric assay (QuantiChrom Heme Assay Kit, BioAssay Systems, USA), so that patients with highest and lowest heme levels were represented in sample. All included SCD patients were in steady state (i.e., at least 3 months from the last vaso-occlusive crisis or red blood cell transfusion), and 8/20 were using hydroxyurea. Whole blood samples were obtained by venipuncture and allowed to clot at room temperature for 30 min, and then centrifuged at 1,000*g* (4°C, 15 min) for serum separation, which was stored at −80°C until analysis. Subject characteristics were recorded at the time of sample collection. Based on ECIS results from a previous study from our laboratory with sepsis patients, a sample size of 20 patients and 10 controls was planned, to obtain a power of 80% and a type II error rate of 0.05.

### Immunofluorescence

Cells were grown to confluence for 48 h on fibronectin (10 µg/ml) pre-coated microscopy-grade glass coverslips, serum starved for 2 h, and stimulated with heme 30 µM for 6 h. HUVECs were then fixed in paraformaldehyde (4% for 20 min), washed with phosphate-buffered saline (PBS), treated (5 min) with 10 mM glycine, permeabilized with 0.2% Triton-X in PBS, rinsed and blocked with 3% bovine serum albumin in PBS (15 min). Cells were then incubated with anti VE-cadherin antibody (30 min), rinsed and incubated with Alexa Fluor-coupled secondary antibodies (30 min). Actin filaments were detected with fluorescent phalloidin. Confocal laser scanning microscopy was carried out using a Zeiss LSM 510 microscope, equipped with a 63 × 1.3 oil immersion objective. Intercellular gaps were quantified using Image J, by an investigator blinded to experimental condition, as previously described (38). Briefly, a total of 10 images, each containing approximately twenty cells were analyzed for each experiment. Image contrast was adjusted semi-automatically until saturation, so that areas of the confluent monolayer that yielded no signal in all fluorescence channels could be identified as gaps, and selected by creating a threshold. Then, the proportion of empty areas in respect to total image area was calculated. To show the empty areas, the region obtained with the threshold was blue-colored and flattened to the original image.

### Measurement of Hemopexin and sVCAM-1 Levels

Hemopexin and sVCAM-1 levels were measured in serum by Elisa in accordance with manufacturer’s instructions (Abcam ab171576, Cambridge, UK; and R&D #DY809, Minneapolis, USA, respectively).

### Statistical Analysis

Differences in continuous variables were analyzed using Student’s t-test/Anova or Mann-Whitney/Kruskal-Wallis tests according to: variable distribution (Gaussian or non-Gaussian respectively) assessed by the D’Agostino & Pearson normality test, and to the number of groups in each comparison. Correlation was calculated using the Spearman correlation coefficient. Data are expressed as mean ± SEM or median and range, as specified. A P-value ≤ 0.05 was considered statistically significant. All statistical analysis was performed with GraphPad Prism 7.0 Software (GraphPad Inc., San Diego, CA, USA).

## Results

### Heme Induces a Transient and Dose-Dependent Disruption of EB

**Figure 1:**
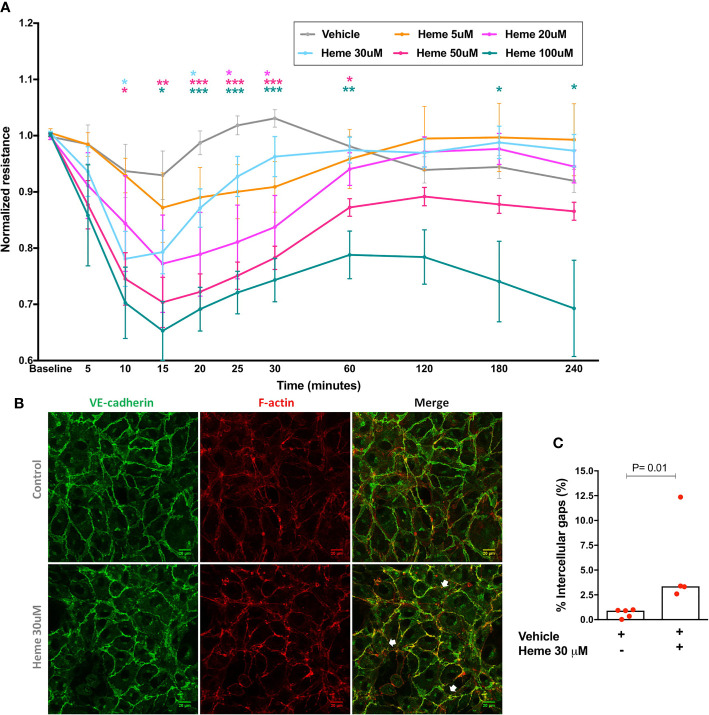
Endothelial barrier integrity after heme stimulation. **(A)** Each line represents the mean ± SEM of the normalized resistance of HUVECs stimulated with heme in serum-free solutions measured by ECIS at 4,000 Hz. Differences between vehicle and heme-stimulated cells were compared using the Kruskal-Wallis test with the Dunn’s posttest. Statistical significant differences are indicated by colored asterisks on each timepoint. *P= 0.01 to 0.05; **P<0.01; ***P< 0.001; n = 5 to 15 independent experiments in at least three different days per heme concentration. **(B)** HUVECs were treated for 6 h with vehicle or heme 30 µM, and stained for the cell-cell junction marker VE-cadherin and for filamentous actin (F-actin). White arrows indicate intercellular gaps. **(C)** Semi-automated image processing identified intercellular gaps in the images that were quantified respect to the total area of the cell monolayer (n= 4 to 5 per group in two independent experiments). Mann-Whitney test; *P =0.01. Vehicle corresponds to the same solution used to dilute heme (NaOH 0.1 M), without heme. The final NaOH concentration in each heme dilution varied from 0.01 to 0.2 µM for heme 5 µM to heme 100 µM. In vehicles, higher NaOH concentrations were used (0.06 to 0.2 µM in panel **A**, and 0.06 µM in panel **B**).

We first demonstrated that heme is capable to induce a dose-dependent disruption of EB in HUVECs, which attains statistical significance as early as 10 min after stimulation, and that persists for 30 min with heme concentrations between 20 and 30 µM, for 60 min with heme 50 µM, returning to baseline values after these timepoints except for heme 100 µM concentration (**Figure 1A**). Of note, the effect vehicle (NaOH) caused no changes in EB integrity measured by ECIS (**Figure S1A**). In order to confirm if these functional changes were associated with morphological changes, we selected a representative heme concentration (30 µM) to stimulate HUVECs, which increased intercellular gap counts in VE-cadherin stained slides (**Figures 1B, C**).

### Sera From SCD Patients Cause EB Disruption *In Vitro*

**Figure 2:**
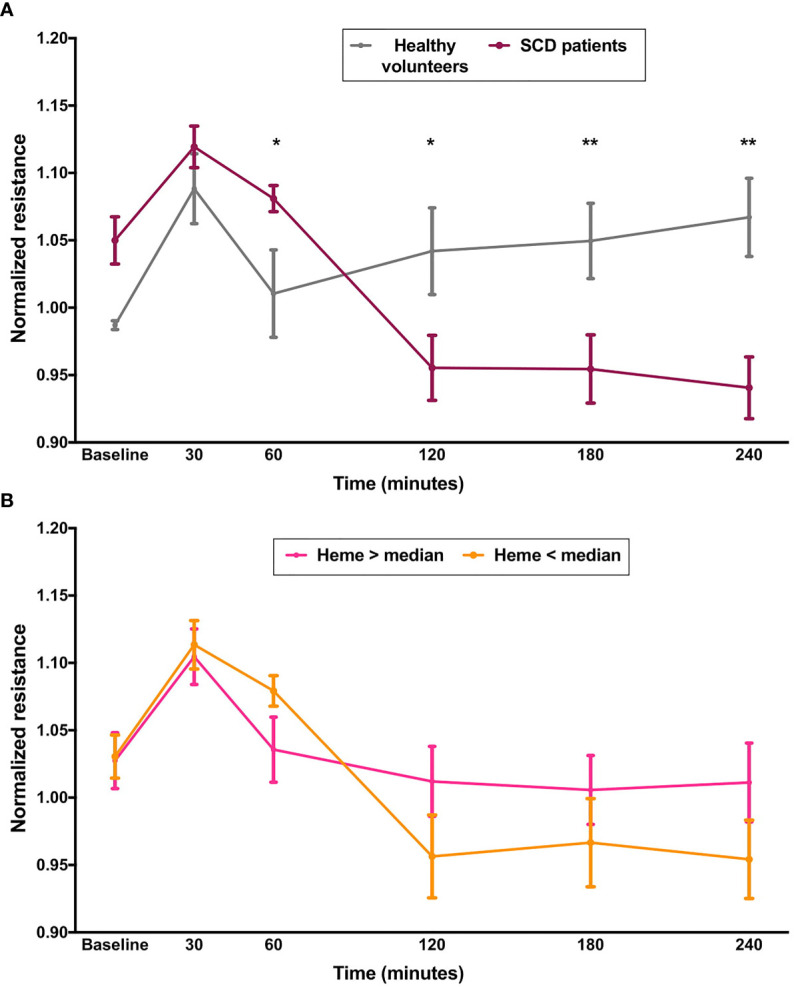
Effect of sera from SCD on EB integrity. Confluent HUVEC monolayers were incubated with sera (20% v/v) from SCD patients (n=20) or healthy volunteers (n=10) and normalized resistance was recorded at 4,000 Hz. Each line represents the mean ± SEM in the specified time points from experiments comparing **(A)** patients and healthy volunteers; or **(B)** patients subgrouped by total levels of heme in serum. Differences were compared using the Mann-Whitney test. Statistical significant differences are indicated by colored asterisks on each timepoint. *P= 0.01 to 0.05; **P<0.01.

Next, we investigated whether sera from patients with SCD, containing varying levels of heme, could elicit changes in EB integrity in HUVECs. Characteristics of our study population are shown in **Table S1**. First, we compared the effect of sera from all SCD patients with those from healthy volunteers. As shown in **Figure 2**, after a period of EB stability observed in both groups, SCD sera elicited a significant decrease in normalized resistance, consistent with EB disruption (**Figure 2A**). This effect persisted for at least 4 h. We then separated patients in two groups according to median levels of total heme in serum (63.0 µM). However, no differences could be observed between these two patient subgroups (**Figure 2B**). It should be noted that while statistically significant, the effects of serum presented a lower magnitude than the effects of heme in aqueous solutions. Of note, no difference could be observed between HU users and non-users (**Figure S1B**).

### Sera From Healthy Volunteers, but Not From SCD Patients, Inhibit EB-Disrupting Effects of Heme

**Figure 3:**
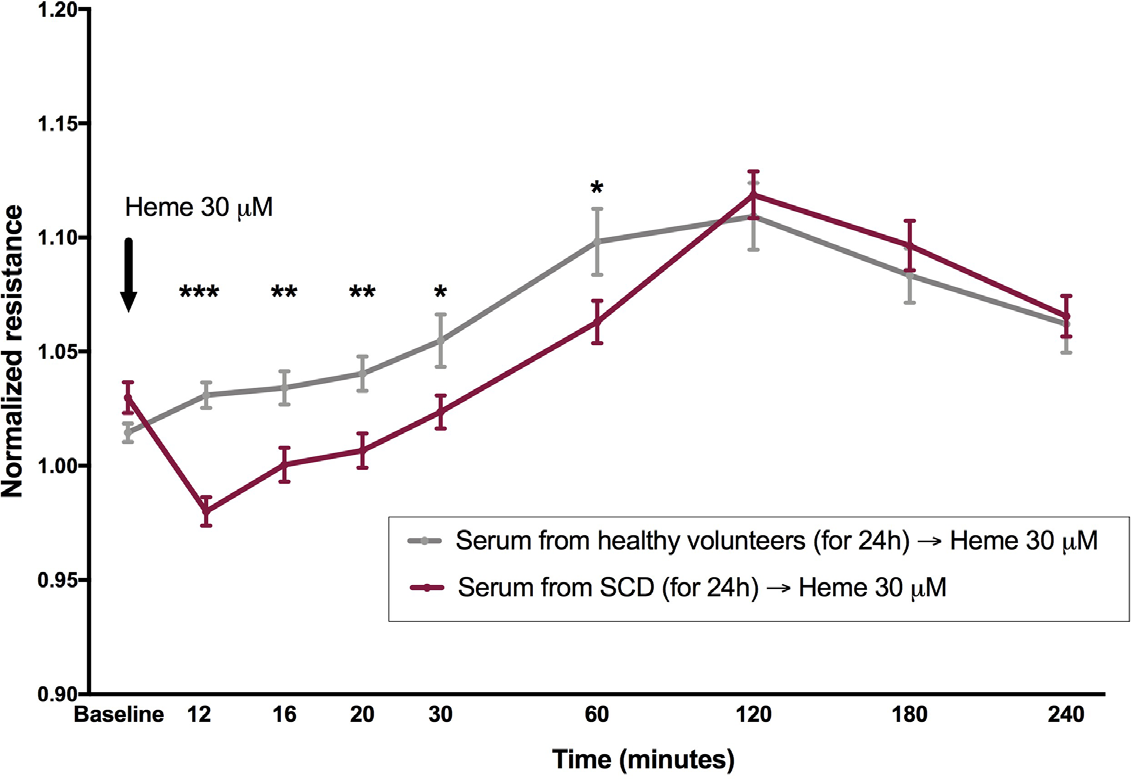
Heme is capable to induce EB disruption in the presence of serum from patients with SCD, but not from healthy volunteers. Confluent HUVEC monolayers were incubated with sera (20% v/v) from SCD patients (n = 20) or healthy volunteers (n = 10) for 24 h, followed by challenge with heme 30 µM (black arrow). Normalized resistance was recorded at 4,000 Hz. Each line represents the mean ± SEM in the specified time points. Differences were compared using the Mann-Whitney test. Statistical significant differences are indicated by colored asterisks on each timepoint. *P= 0.01 to 0.05; **P<0.01; ***P<0.001. Heme was diluted in NaOH, and the final NaOH concentration in heme dilutions was 0.06 µM.

Since total serum heme (which includes mainly the protein-bound fraction of this molecule) was not a significant determinant of the magnitude of EB disruption, we hypothesized that an acute increase in heme levels would be necessary to reproduce the effect of heme shown in **Figure 1**, based on the assumption that FEH might not be available in a protein-rich matrix such as serum. In order to test this hypothesis, cells were incubated with serum from healthy volunteers or SCD patients for 24 h in ECIS arrays, and then challenged with heme to a final concentration of 30 µM. While the presence of sera from healthy volunteers prevented the effects of heme on EB integrity, a milder, yet significant disruption of EB was observed when heme was added to cells incubated with sera from SCD patients (**Figure 3**).

### Heme-Induced EB Disruption Is Associated With Hemopexin Levels

**Figure 4:**
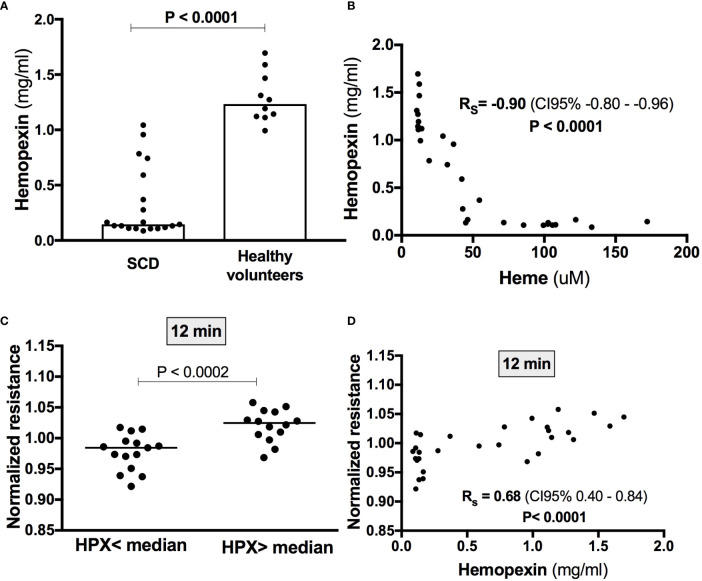
Heme-induced EB disruption in the presence of serum is associated with hemopexin levels. **(A)** Hemopexin levels were measured by Elisa and were lower in SCD patients (n=19) compared to healthy volunteers (n=10) (Mann-Whitney test). **(B)** A strong negative correlation was observed between hemopexin and heme levels (Spearman correlation coefficient). **(C)** Lower values of normalized resistance at the timepoint of peak heme-induced EB disruption were observed in individuals with lower hemopexin levels (t test). Accordingly, peak heme-induced EB disruption (i.e., after 12 min) was statistically correlated with hemopexin levels (Spearman correlation coefficient) **(D)**.

Since free heme is quickly removed from the circulation by hemopexin, we hypothesized that the induction of EB disruption by the addition of heme to SCD sera could be associated with lower hemopexin levels when compared to healthy volunteers. In fact, hemopexin levels were significantly lower in SCD patients compared to controls (0.33 ± 0.32 vs 1.29 ± 0.23; P< 0.001) (**Figure 4A**). As expected, a strong correlation (R_s_ = 0.90; P < 0.0001) was observed between heme and hemopexin levels (**Figure 4B**). Interestingly, when all participants were divided according to median hemopexin levels (0.59 mg/ml), lower values of normalized resistance (which indicate heme-induced EB disruption) were observed in individuals with lower hemopexin levels (**Figure 4C**). Moreover, the magnitude of heme-induced EB disruption at 12 min (the timepoint when this effect was more evident) were correlated with hemopexin levels (R_s_ = 0.68; P < 0.0001) (**Figure 4D**). Of note, a strong correlation was observed between hemopexin and sVCAM-1 (R_s_ = −0.72; P< 0.001). However, while levels of sVCAM-1 were also associated with normalized resistance, the correlation coefficient was weak (Rs = −0.42; P = 0.03).

## Discussion

EB disruption is a hallmark of several inflammatory diseases (39–42), and studies in animal models suggest that this process is involved in the pathogenesis of acute complications of SCD, namely ACS (21, 22). Accordingly, the effects of hemolysis byproducts such as free hemoglobin and heme on EB integrity in endothelial cell monolayers have been recently investigated, with consistent data supporting an EB-disrupting effect of heme (24–26). However, due to the strong affinity of heme to circulating proteins, coupled with the possible interplay of heme with other inflammatory mediators, it is important to confirm these effects in experimental conditions that more closely resemble those observed in the clinic. The most important result of our study was the demonstration that heme is capable to induce EB disruption even in the presence of serum proteins, but that this effect only occurs with serum from SCD patients, but not from healthy volunteers, and is associated with hemopexin levels.

Based on the demonstration that heme can trigger innate immunity activation (1, 13–15), excess heme was associated with the pathogenesis of vaso-occlusion (43) and ACS (22, 44) in SCD. Accordingly, a growing interest emerged on whether alterations of the alveolar-capillary barrier participated in the pathogenesis of ACS, as well as if heme was capable to induce EB disruption. In regard to the former, studies using mice models of SCD suggested that EB disruption was involved in the pathogenesis of ACS (21, 22). Moreover, isolated pulmonary endothelial cells from homozygous sickle mice (SS) were shown to be more sensitive to the EB-disrupting effects of LPS (measured by ECIS) than cells from heterozygous (AS) mice (45). In regard to the latter question, the first demonstration that heme could elicit EB disruption was published almost 20 years ago in a study that demonstrated the accumulation of radiolabeled liposomes in different organs of C57Bl/6 mice treated with heme (46), a finding that was also demonstrated using other *in vivo* assays of EB integrity in mice (11). In the last 4 years this observation was confirmed and further explored in cell-based assays, in which the exposure of pulmonary or microvascular endothelial cells to heme (diluted in aqueous solutions of NaOH) consistently induced EB disruption in both static and flow-based (microfluidic) assays (24–26). These studies also demonstrated that these effects occurred in the context of the effects of heme on innate immunity, since they were TLR4-dependent. However, one of the caveats of studies about the effects of heme on innate immunity is the strong affinity of this molecule for proteins that are abundant in serum such as hemopexin and albumin, so that some authors recently questioned their biological relevance at all (27). Of note, all of the recent cell-based studies about the effects of heme on EB disruption used heme diluted in protein-free solutions.

In our study we first confirmed that heme can disrupt EB, in a dose dependent (in our system, in concentrations above 10 µM) and transient fashion. Barrier integrity returned to normal within 25 to 60 min in cells exposed to the lower range of heme concentrations used in our study (below 30 µM), which more closely resemble the concentrations of FEH in contact to cells in humans. This transient nature indicates that heme-induced barrier disruption is not caused by cell death, and raises the question on which of the signaling pathways involved in the regulation of EB integrity are modulated by heme. This observation is also consistent with a previous study that also showed a dose-dependent effect of heme on EB integrity, and that showed that heme used at a higher concentration (40µM) was associated with a more delayed disruption of EB integrity that was attributed to necroptotic cell death (25), but other pathways that are modulated by heme such as autophagy (47–49) and MKK3/p38MAPK (50) have also been recently associated with EB changes. It should be noted that our results also confirm that the EB of HUVECs behave similarly to pulmonary and microvascular endothelial cells in response to heme, supporting their use in our subsequent experiments.

We also demonstrated that sera from patients with SCD, but not from healthy volunteers, induce EB disruption of HUVEC monolayers. Regulation of EB integrity is a complex process that involves cellular and humoral mediators (51–53), both altered in SCD. In this regard, our results suggest that soluble inflammatory mediators contribute at least in part to EB disruption in SCD, and that their identification could generate important insights about the pathogenesis of this disease. Given the complex nature of this process, high-throughput strategies such as proteomics or metabolomics would possibly be more adequate than the testing of isolated candidate modulators by immunological methods. As far as we are aware, only one group studied evaluated the effect of specific plasma components on EB integrity in SCD. This study showed that exosomes from SCD patients with a higher frequency of ACS (mainly derived from red blood cells), induced a more pronounced disturbance of the EB on human microvascular endothelia cells than exosomes from patients with no history of ACS (54). Of note, the same group had previously shown that these exosomes were mainly derived from endothelial cells, and had a miRNA cargo capable to discriminate mild from severe clinical phenotypes (55). As in our study, all patients from the former study were in steady-state when samples were collected, and EB function was measured by ECIS. Since our study was focused on the role of heme as an EB-disrupting agent in SCD, we first investigated whether total heme levels in these serum samples influenced the magnitude of EB disruption, which was not confirmed. Our negative results can be probably explained by the fact that total heme levels encompass not only FEH, but mainly heme bound to hemopexin, albumin and hemoglobin (56), which is not capable to activate the immune system. Accordingly, it is likely that levels of FEH in stored serum samples are extremely low, or even absent, as suggested by others (56). So, we added heme to cells in the presence of serum to investigate whether an acute increase in serum heme concentrations could reproduce the effects of heme on EB. By doing this, we were able to show that proteins present in serum from patients with SCD are not sufficient to inhibit the EB-disruption induced by an acute challenge with heme. Interestingly, addition of heme to sera from healthy volunteers did not elicit any effect on EB, suggesting that the inflammatory milieu characteristic of serum from SCD patients is required for heme-induced EB disruption.

Of all heme scavenging proteins present in serum, hemopexin is the one with the highest affinity, recognized as a critical line of defense against FEH. As expected, hemopexin levels were markedly lower in SCD patients compared to healthy volunteers. Moreover, hemopexin levels were also correlated with both heme and sVCAM-1, which is a marker of endothelial activation in SCD. Interestingly, we demonstrated that peak heme-induced EB disruption in the presence of serum was associated with hemopexin levels, which as far as we are aware represent the first time when human hemopexin levels were associated with modulation of EB function. Together these results provide additional support for the concept that FEH can directly contribute to EB disruption in SCD. Of note, in a recent study, the mechanisms by which heme disrupts the EB were further elucidated and shown to involve endothelial cytoskeleton remodeling (50), paving the way for the study of these pathways in SCD. Finally, our results also demonstrate that the effects of heme on endothelial cells are not restricted to serum free conditions.

Our study has limitations that need to be acknowledged. First, although the ECIS method has been validated as a method to assess the integrity of intercellular junctions by both empirical data (35, 36), as well as by its use in the description of several key features of endothelial barrier function (57–61), it only evaluates the response of endothelial cells to a discrete stimulus, compared to the much more complex regulation of endothelial function *in vivo*. On the other hand, this very characteristic represents an advantage to answer focused research questions such as the one from our study. Another limitation is the use of HUVECs as opposed to other adult organ-specific endothelial cell types, since phenotypic differences have been reported between different endothelial cell types. It should be noted however, that HUVECs have been a valuable tool for studies of vascular phenotype for several decades, including studies about central aspects of endothelial barrier biology during inflammation (62, 63). In addition, in the first part of our study using heme in NaOH solutions we demonstrated that HUVECs respond to heme in a similar fashion compared to pulmonary and microvascular endothelial cells in regard to EB function. We should also mention that although our results demonstrate a yet unknown association of serum hemopexin levels with heme-induced *in vitro* EB disruption in SCD, this association does not allow us to claim for a causal relationship between hemopexin deficiency and EB disruption, which requires additional studies investigating whether hemopexin can reverse these changes. Another limitation of our study is related to the fact that methods used to measure heme in most studies involving SCD are not capable to separate total or cell free heme, from protein-free heme (i.e., not bound to hemopexin, albumin or other proteins). This fraction, referred in our study as FEH, is the one that is expected to be toxic to cells and tissues. This methodological limitation could explain why total heme levels were not associated with EB disruption, whereas hemopexin levels, which is consumed by the release of free heme, were. Studies using recently described methods capable to measure protein-free heme (64, 65) are warranted to address this issue. Another limitation that deserves to be discussed is the relevance of adding NaOH-solubilized heme to cell cultures, as a model of heme release. In fact, important details about the kinetics of heme release from damaged red blood cells in patients with hemolytic disorders are yet to be clarified, and the very existence of FEH *in vivo* has been discussed (27). Although the role of red blood cell microparticles as mediators of heme transfer has been recently demonstrated (66), one cannot exclude that other aspects of the interaction of heme with blood components that are not included in our model may be key to its pathological effects *in vivo*. Nevertheless, we believe that our strategy of adding heme to patient serum, and measuring EB function in real time can overcome at least some of these limitations, potentially representing a closer model to the pathological effects of heme *in vivo.* Since all our experiments were performed with serum, we should also mention the fact that some of the pro-inflammatory effects of heme in cell models were only observed in serum free conditions (1). Finally, the relatively low sample size of our study should also be considered when interpreting our results.

In conclusion, we demonstrated that the previously described transient disruption of EB by heme is also observed in the presence of sera from SCD patients, corroborating the role of FEH in the pathogenesis of this condition, through the demonstration that its effects are not restricted to serum free conditions. The fact that the effects of heme on EB are only observed in serum from SCD and that this effect is associated with hemopexin levels support the concept that heme could directly contribute to EB disruption in SCD, thus warranting additional studies to confirm this causal relationship. Together our *in vitro* studies provide additional support to the concept that heme may act as a danger-associated molecular pattern (DAMP) in hemolytic conditions.

## Data Availability Statement

The datasets generated for this study are available on request to the corresponding author.

## Ethics Statement

The studies involving human participants were reviewed and approved by Institutional Review Boards HEMOPE (protocol 510.517) and of University of Campinas (protocol 3.291.418). The patients/participants provided their written informed consent to participate in this study.

## Author Contributions

VS and MF designed the study, performed endothelial cell assays, and analyzed data. LC performed endothelial cell assays. DG-W revised and analyzed confocal data. BH and FC contributed to study design and measured heme levels. WT and FL contributed to hemopexin and sVCAM-1 measurements. ID, AA, AL-A, MB, and MN recruited and monitored patients. IB-J performed ECIS studies with NaOH and thrombin. FFC contributed with reagents and laboratory infrastructure. JM supervised ECIS experiments and contributed with reagents and laboratory infrastructure. EP designed the study, reviewed, and analyzed data and drafted the manuscript. All authors contributed to the article and approved the submitted version.

## Funding

This study was funded by the Sao Paulo Research Foundation (FAPESP), grants 2016/14172-6 to EP, and 2014/00984-3 to FFC, respectively; Conselho Nacional de Desenvolvimento Cientifico e Tecnologico (CNPq) Brazil; Coordenacao de Aperfeicoamento de Pessoal de Nivel Superior – Brasil (CAPES), finance code 001; and FAEPEX-UNICAMP.

## Conflict of Interest

The authors declare that the research was conducted in the absence of any commercial or financial relationships that could be construed as a potential conflict of interest.

## Supplementary Material

Supplementary Figure 1 Exploration of the effects of NaOH and hydroxyurea (HU) on EB. In **(A)**, different concentrations of NaOH, which was the vehicle used to dilute heme, were used under the same experimental conditions. Each line represents the mean ± SEM of the normalized resistance of HUVECs stimulated with either NaOH or thrombin used as a positive control. Normalized resistance was measured by ECIS at 4,000 Hz. As shown in the upper panel, NaOH at different concentrations did not affect EB. In **(B)**, data from SCD patients used in **Figure 3** were subdivided according to the use or not of hydroxyurea (HU). No difference in ECIS readings after heme stimulation could be detected between these two subgroups. Kruskall Wallis and Dunn’s posttest.
