# Supplementary figures and images for: Endothelial Barrier Integrity Is Disrupted In Vitro by Heme and by Serum From Sickle Cell Disease Patients
Source: Front Immunol. 2020 Dec 14;11:535147. doi: 10.3389/fimmu.2020.535147 (PMC7767881; doi:10.3389/fimmu.2020.535147)

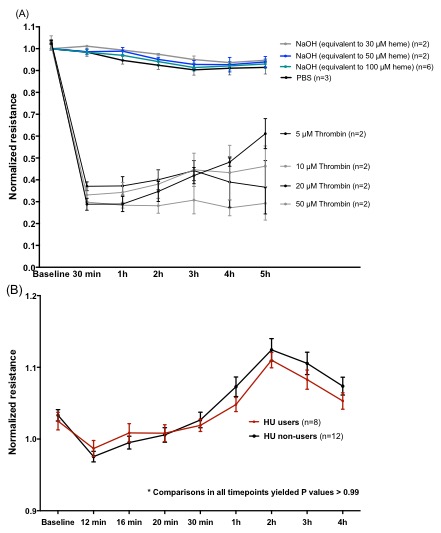

Supplement: Supplementary Figure 1 — Exploration of the effects of NaOH and hydroxyurea (HU) on EB. In (A), different concentrations of NaOH, which was the vehicle used to dilute heme, were used under the same experimental conditions. Each line represents the mean ± SEM of the normalized resistance of HUVECs stimulated with either NaOH or thrombin used as a positive control. Normalized resistance was measured by ECIS at 4,000 Hz. As shown in the upper panel, NaOH at different concentrations did not affect EB. In (B), data from SCD patients used in Figure 3 were subdivided according to the use or not of hydroxyurea (HU). No difference in ECIS readings after heme stimulation could be detected between these two subgroups. Kruskall Wallis and Dunn’s posttest. [file Image_1.jpeg]
