# Supplementary material for: Endothelial Barrier Integrity Is Disrupted In Vitro by Heme and by Serum From Sickle Cell Disease Patients
Source: Front Immunol. 2020 Dec 14;11:535147. doi: 10.3389/fimmu.2020.535147 (PMC7767881; doi:10.3389/fimmu.2020.535147)
Supplement: Supplementary file 2 [file Table_1.pdf]

Table S1. Clinical characteristics of the study population

**Characteristics**	**Patients** (n = 20)	**Healthy individuals** (n=10)
Demographic and laboratory data		
**Age,** median (min-max)	31 (20 – 55)	22 (20 – 26)
**Sex,** male/female	14/6	2/8
**Total heme**, µM, median (min-max)	63.0 (19.2 – 172.2)	11.8 (10.6 – 14.0)
**Hemopexin**, mg/mL, median (min-max)	0.14 (0.09 – 1.04)	1.23 (0.99 – 1.69)
**sVCAM-1**, ng/mL, median (min – max)	791.5 (428.2 - 1356.0)	473.1 (351.4 – 836.4)
**Albumin**, g/dL, median (min – max)	4.2 (3.5 – 5.3)	4.4 (3.6 – 4.9)
**Total protein**, g/dL, median (min – max)	7.4 (6.2 – 9.9)	7.0 (6.1 – 7.7)
Clinical data		
**Hemoglobin**, g/dL, median (min-max)	7.7 (5.3 – 9.6)	13.6 (12.2 – 17.2)
**Reticulocyte count,** %, median (min-max)	11.9 (4.0 – 28.5)	-
**LDH,**U/L, median (min-max)	490 (280 – 1755)	-
**Leukocytes**, $*10^3$/uL, median (min-max)	10.9 (7.1 – 23.8)	7.7 (4.6 – 10.1)
**Neutrophils**, $*10^3$/uL, median (min-max)	6.4 (2.6 – 16.7)	4.6 (1.4 – 7.2)
**Monocytes**, $*10^3$/uL, median (min-max)	1.2 (0.5 – 3.1)	0.5 (0.1 – 0.7)
**Platelets**, $*10^3$/uL, median (min-max)	370.0 (177.0 – 812.0)	255.5 (170.0 – 390.0)
**Hydroxyurea use,** n (%)	8 (40%)	-

Table S2. Albumin and total protein levels according to hydroxyurea (HU) use

	**HU users** (n = 8)	**HU non-users** (n=12)	**P***
**Albumin**, g/dL, median (min – max)	4.2 (3.5 – 5.3)	4.3 (3.9 – 4.9)	0.13
**Total protein**, g/dL, median (min – max)	7.3 (6.2 – 9.9)	7.6 (6.9 – 8.9)	0.92

* Mann-Whitney test
